# From Amateur to Professional Cycling: A Case Study on the Training Characteristics of a Zwift Academy Winner

**DOI:** 10.3390/sports13070234

**Published:** 2025-07-16

**Authors:** Daniel Gotti, Roberto Codella, Luca Vergallito, Andrea Meloni, Tommaso Arrighi, Antonio La Torre, Luca Filipas

**Affiliations:** 1 Department of Biomedical Sciences for Health, Università degli Studi di Milano, 20122 Milan, Italy; daniel.gotti@unimi.it (D.G.); luca.vergallito@gmail.com (L.V.); andreameloni.cp@gmail.com (A.M.); t.aarrighi@gmail.com (T.A.); antonio.latorre@unimi.it (A.L.T.); luca.filipas@unimi.it (L.F.); 2 Department of Endocrinology, Nutrition and Metabolic Diseases, IRCCS MultiMedica, 20138 Milan, Italy; 3 TotalEnergies Pro Cycling Team, 85140 Essarts-en-Bocage, France

**Keywords:** cycling performance, training periodization, exercise adherence, public health implications of virtual cycling, endurance training strategies, Zwift Academy (ZA)

## Abstract

This study aimed to describe the training leading to the Zwift Academy (ZA) Finals of a world-class road cyclist who earned a professional contract after winning the contest. Four years of daily power meter data were analyzed (male, 25 years old, 68 kg, VO_2_max: 85 mL·min^−1^·kg^−1^, and 20-min power: 6.37 W·kg^−1^), focusing on load, volume, intensity, and strategies. Early training alternated between long, moderate-intensity sessions and shorter high-intensity sessions, with easy days in between. Gradually, the structure was progressively modified by increasing the duration of moderate-intensity (MIT) and high-intensity (HIT) and, subsequently, moving them to “high-volume days”, creating a sort of “all-in days” with low-intensity (LIT), MIT, and HIT. Moderate use of indoor training and a few double low-volume, low-intensity sessions were noted. These data provide a deep view of a 4-year preparation period of ZA, providing suggestions for talent identification and training, thereby highlighting the importance of gradual progression in MIT and HIT.

## 1. Introduction

Professional cycling is a highly demanding sport that requires a wide blend of physical, psychological, and tactical abilities. Cyclists must excel in several key areas, including aerobic capacity, muscular strength, endurance, and power output, which are essential for success in all types of races, from grand tours to one-day racing [1]. Beyond physical attributes, both psychological and technical factors, such as mental resilience, the ability to perform under pressure, and driving confidence, are equally fundamental [2,3]. Demands of racing and required cyclist attributes in the whole range of levels from Under23 (U23) to World Tour (WT) have been investigated so far [4,5,6], clearly defining the standards for competing on a global level, such as extreme aerobic capacity, high sustained power output, and strong anaerobic capabilities, depending on the type of cyclist and races [7,8].

The level of road cycling has constantly increased in the last three decades due to technological devices, bike quality, and training features. Furthermore, there have been several situations where talents have emerged from other sports without following the standard path. World cycling teams have faced the challenge of employing sophisticated strategies to identify and nurture new talent, ensuring a steady influx of promising young riders into the professional ranks. The process involves a combination of scouting, performance analysis, and developmental programs aimed at discovering and cultivating riders who exhibit the potential to excel in the demanding world of professional cycling [9,10]. Nevertheless, some talents may not be captured by traditional selection methods, creating the need for an alternative “recognition path”. In this context, the need to find new strategies for scouting new athletes became evident.

Zwift Academy (ZA), launched in 2016, has revolutionized the way athletes can break into the professional cycling world by providing an accessible, data-driven pathway from the comfort of their own homes. The platform used in this competition is Zwift (Zwift, 2014, Long Beach, CA, USA), which initially gained popularity as a virtual training tool, allowing cyclists and runners to train indoors while interacting with a global community. Subsequently, during the COVID-19 pandemic, the use of this platform and the consequent participation in the contest underwent a rapid increase, becoming a hotbed of new talents who are still participating in the best world competitions today [11].

In ZA, riders’ performances are monitored and analyzed, allowing coaches and talent scouts to evaluate their potential. As the academy progresses, participants are gradually narrowed down through performance evaluations and competitions until a small group of finalists is selected. The culmination of Zwift Academy is an intense final phase, where the top performers are given the opportunity to train and compete with professional teams. The ultimate prize is a professional contract with a leading cycling team, such as Alpecin–Deceuninck (men’s team) or Canyon//SRAM Racing (women’s team). Although both the characteristics of the races and the abilities of pro cyclists have been clearly reported, to the best of our knowledge there is no long-term analysis of “how” a non-professional cyclist reaches the level of a top world team, especially through a revolutionary route such as a ZA.

The aim of this study is to investigate the training characteristics of a male Zwift Academy winner by examining periodization and the impact of training methodologies. To the best of our knowledge, no study has analyzed a ZA winner, and detailed day-by-day training analyses over such a long period are currently lacking in the literature, which predominantly focuses on short-term adaptations. This study seeks to identify the key traits that have allowed an amateur to become a new pro cyclist.

Although this study focuses on elite performance, the training strategies (e.g., balance between low-intensity training (HIT) and moderate-intensity training (MIT) and indoor cycling integration) could also inform public health guidelines for promoting physical activity in recreational athletes.

## 2. Materials and Methods

### 2.1. Participant

One male professional road cyclist (age: 25 y; body mass: 68 kg; height: 190 cm; relative maximum oxygen consumption: 85 mL·min^−1^·kg^−1^; and relative 20-min record power output: 6.37 W·kg^−1^)—who earned his professional status after winning the ZA—is the subject of this study. The study design and procedures were approved by the research ethics committee of the Università degli Studi di Milano (approval number 52/20, attachment 4) and followed the ethical principles for medical research involving human participants set by the World Medical Association Declaration of Helsinki. The participant provided informed written consent.

### 2.2. Experimental Design

Daily data of 2040 training sessions were selected and analyzed; the timeframe corresponds to the 4 years preceding the Zwift Academy Finals (from 1 October 2018 to 10 November 2022). The COVID-19 pandemic, injuries, rest period, and amateur race schedule of the athlete were reported. The training sessions were categorized as indoor and outdoor, and multiple daily sessions were highlighted. A limit of 50 kJ/kg was used to separate low- and high-load sessions, as suggested in a recent study [12].

### 2.3. Data Processing

Power output from training and races was daily collected using portable power meters (Power2max Type S) that were zeroed before every ride. The accuracy and precision of this power meter were reported in a previous study [13]. Data were saved and organized using a cycling performance software analyzer (WKO5, TrainingPeaks LLC, Louisville, CO, USA). All data were visually inspected to identify any errors or incomplete files, which could have been caused by technical issues (e.g., a flat battery in a power meter).

Inclusion criteria for power data required that the sum of time spent in power zones accounted for at least 80% of the daily total training volume. This threshold was set to ensure an accurate representation of each training session. When the sum of time spent in power zones was >80% but did not reach 100% of the daily total volume, time in each power zone was adjusted proportionally. Despite this cautious threshold, the dataset retained over 95% of the original data.

### 2.4. Volume and Intensity Distribution

Volume was considered as the duration of the training/racing session. Intensity distribution was calculated using a three-zone power-based model; to align with the real training strategies employed during the preparation for the Zwift Academy Finals, data from First Lactate Threshold (LT1) and Second Lactate Threshold (LT2)—boundaries between zone 1–2 and zone 2–3, respectively [14,15]—were determined using field tests and updated periodically based on the results of new assessments. Different testing methods were employed with reasonable frequency and independently conducted, including critical power (CP) tests over various durations, constant-power tests with lactate sampling to identify LT1, and incremental on-road tests for lactate profiling. To validate these training zones, a double-check was conducted against the athlete’s recorded power output (RPO)—the best performances achieved over specific time intervals—and results from cardiopulmonary exercise testing (CPET) in a laboratory setting performed at different moments during the 4 years—RPO consistently, CPET June 2021 and February 2022. The thresholds were confirmed to be appropriate. Time spent in zone 1 was considered as low-intensity training (LIT), time in zone 2 as moderate-intensity training (MIT), and time in zone 3 as high-intensity training (HIT) [16]. The polarization index (PI) was also used to describe the training intensity distribution adopted by the cyclist; it is calculated as PI = (% Time LIT + % Time HIT)/% Time MIT. This index reflects the degree of polarization in training intensity, indicating the balance between predominantly low- and high-intensity efforts versus moderate intensity [17].

### 2.5. Statistical Analysis

Descriptive statistics for the athlete were used. For each variable, mean, standard deviation, maximum, minimum, and monthly values were reported.

## 3. Results

Training and race characteristics of the 4 years are reported in Table 1.

**Figure 1:**
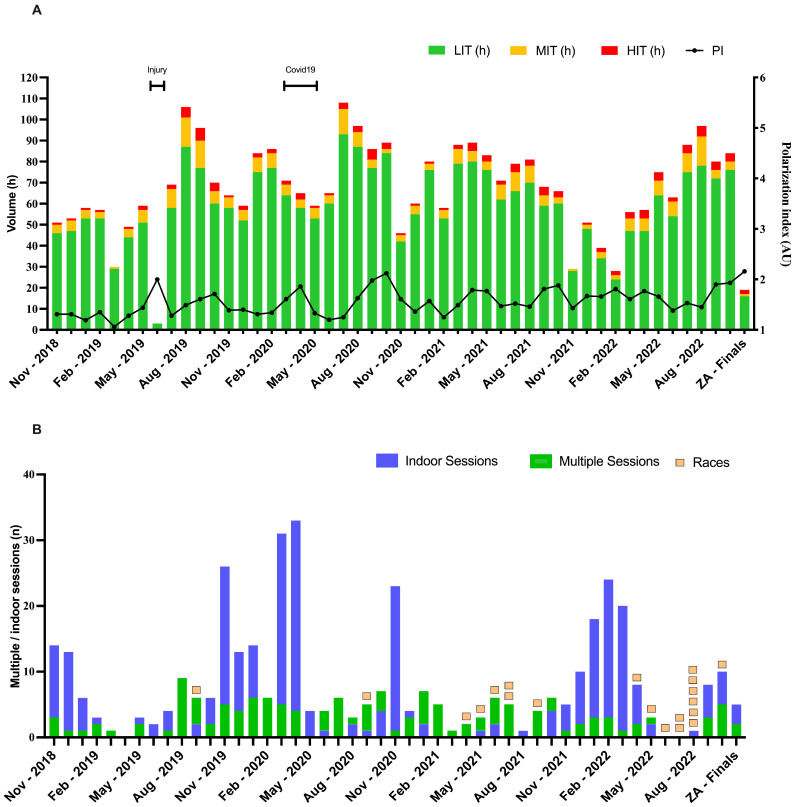
(**A**) LIT (low-intensity training), MIT (moderate-intensity training), HIT (high-intensity training), and PI (polarization index) during the 4 years. The sum of the three zones reflects overall volume. (**B**) Indoor sessions, multiple trainings, and races in the 4 years of preparation.

**Figure 2:**
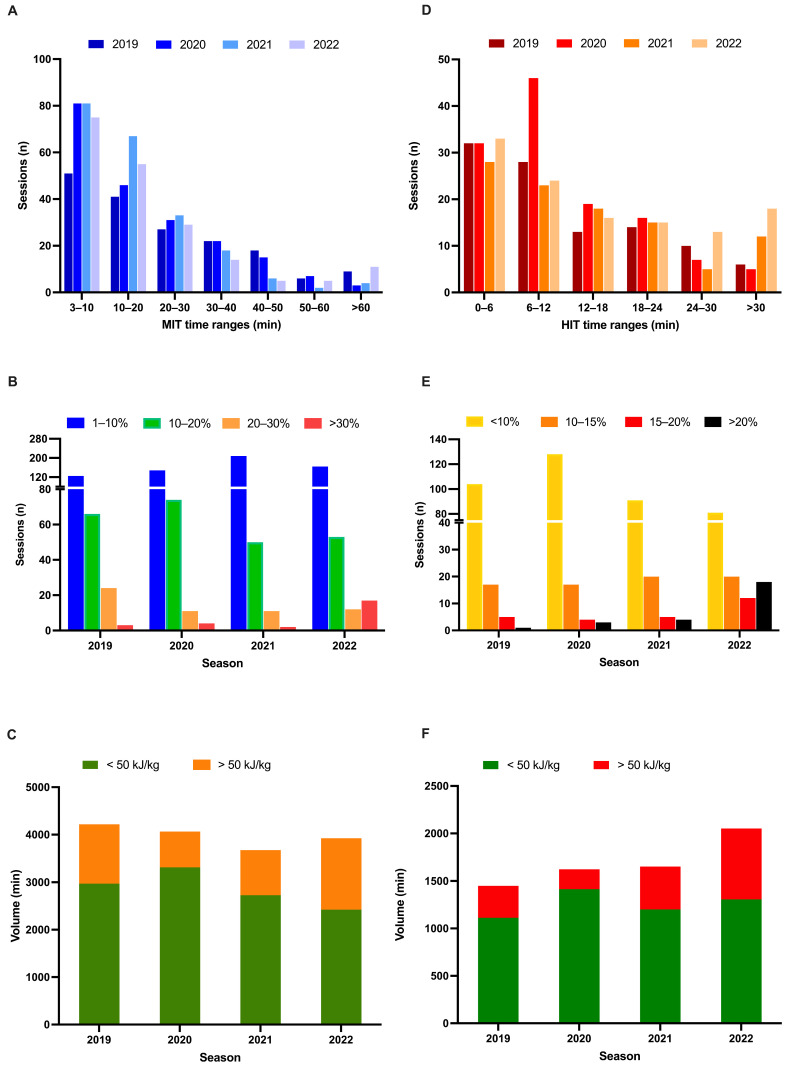
(**A**) Total number of MIT sessions across seasons categorized by duration. (**B**) Total number of sessions categorized by % of time spent in MIT. (**C**) Positioning of MIT volume categorized by KJ consumed, with boundary placed at 50 kJ/kg. (**D**) Total number of HIT sessions across seasons categorized by duration. (**E**) Total number of sessions categorized by % of time spent in HIT. (**F**) Positioning of HIT volume categorized by KJ consumed, with boundary placed at 50 kJ/kg.

Volume and intensity distribution, with details on multiple sessions and races, are highlighted in Figure 1. Deeper analyses of MIT and HIT management are reported, respectively, in Figure 2.

## 4. Discussion

### 4.1. Volume and LIT

The weekly and monthly training volume over the past 4 years has followed a similar wavelike pattern due to seasonal and lifestyle rhythms, ranging from 50 to 110 h, for most of the time similar to the average seasonal volume for both World Tour riders and Grand Tour top-5 finishers (~20 h/week) [5,18,19]. The highest volume occurred, on average, between July and September of every year, identifiable as an “endurance block”. However, certain events such as COVID-19 [20] or injuries affected the volume for several months, causing unusual drops. This non-linear fluctuation likely had a positive effect, preventing steady-state conditions and reducing training monotony [21]. The training volume, especially at low intensity, aligns with that of a world-class cyclist, covering a solid 89 ± 4% of total time, providing a stable foundation to sustain the essential sport-specific physiological stimulus [22].

### 4.2. MIT

The overall time spent in this zone is consistent across all 4 years, ranging from 60 to 70 h per year, and represents approximately 8% of the total training time, largely compatible with the volumes reported in other similar studies [18,19]. Additionally, the average weekly load is proportional to the total volume within the same time frame, reflecting the average monthly trend of the polarization index, which rarely exceeds 2.0. These data confirm the difficulty of achieving a true polarized distribution during training periods for high-volume athletes [18,19].

Upon closer examination, we observe that the time spent in MIT during each session has evolved over the 4 years. While the number of workouts with a maximum of 40 min of MIT remains similar, the last training year preceding ZA Finals showed a noticeable increase in sessions lasting over an hour, indicating a marked increase in high-volume, medium-intensity days (Figure 2A). This observation is further supported by Figure 2C, which highlights that despite the total time spent in the MIT zone being unchanged, the year preceding Zwift Academy saw significantly more sessions surpassing 50 kJ/kg as a clear transition to a more structured durability training compared to previous years.

Moreover, the percentage of workouts with over 30% of total time dedicated to MIT has risen annually, reaching a level nine times higher in the last year than the year prior. This suggests a shift towards a greater combination of volume and intensity in the final phase of preparation by progressively simulating the physiological demands of specific events, which now more than ever emphasize the concept of durability, recently defined as the fourth dimension of endurance [7,23,24]. Finally, given the increase in time spent and the number of threshold intervals performed, this athlete reflects the inherent challenge of adhering to a truly polarized model during specific periods of the year [25,26].

### 4.3. HIT

Although HIT appears to remain constant throughout the years from a monthly perspective, its distribution varied considerably throughout the entire period. The time spent in zone 3 during each workout followed the MIT trend, increasing annually and peaking in the final year with the highest number of workouts lasting more than 30 min (Figure 2D). In the third year before ZA, a noticeable peak occurred in the 6–12 min range, indicating the use of a microdosing strategy, which was not continued in subsequent seasons. This pattern appears to align with the principle that an excess of HIT may not be beneficial, leading to its reallocation within training sessions rather than a continuous increase. In addition, redistributing HIT rather than exceeding tolerance ranges could be useful to prevent injury while still providing an adaptive stimulus [27,28]. In addition, we observed a reduction in the number of days with less than 10% of time spent above LT2 during the last 2 years near ZA, favoring sessions where more than 20% of the time was spent above LT2—up to five times more than in previous years (Figure 2E).

Finally, the relationship between volume and HIT shows a significant increase in time spent above 50 kJ/kg during training, highlighting the clear use of high-volume, high-intensity training days over the years following the current race’s key factors and the demands of ZA Finals that include RPO tests, all-out climbs, and sprints [8]. In retrospect, the approach followed over the four years appears to have led to excellent adaptation in terms of repeatability while avoiding the negative effects of excessive reliance on high-intensity training (HIT) [28].

### 4.4. Indoor and Multiple Sessions

The number of indoor sessions showed three main peaks: the first at the end of winter 2020, driven by the COVID-19 pandemic; the second in the following November; and the final peak in the winter of 2021–2022, due to weather conditions and limited time availability (Figure 1B). Beyond this period, the use of the home trainer was minimal but consistent, with a small pre-competition block toward the end to ensure adaptability to the indoor setting of the ZA, which required a moderate level of specificity due to the different pedaling mechanics [29,30,31]. The use of multiple sessions followed a more flexible, day-to-day approach. The second daily session was introduced primarily to accommodate personal mobility needs or indoor workouts without following a structured periodization plan.

### 4.5. General Trends and Training Characteristics

Firstly, training was prescribed by the athlete himself in consultation with different coaches over the years, following a flexible and pragmatic approach. Elements from various training methodologies were selectively adopted according to individual goals and evolving circumstances. The overall structure of the athlete’s training over the last four years leading up to the ZA finals was influenced by several factors, with time availability playing a significant role. As a student, he had more time for training compared to a regular worker, allowing for a consistent average volume. Other lifestyle factors independent of training, such as personal relationships and student choices, also affected the training load. During the summer, high-volume training blocks were common, with the majority of training hours occurring in this period. In colder months, training was primarily conducted outdoors, with the indoor trainer used as an optional tool. Competitions were concentrated in two main periods: April–August 2021 and April–August 2022.

No structured strength or cadence training was implemented during these four years. No specific race calendar was implemented during the period; amateur races were included in the training plan solely for the enjoyment of competition. No specific tapering strategies were identified in the final weeks leading up to the ZA finals, apart from a subjective pursuit of freshness, supporting the trend of tailoring strategies based on individual preferences. Consistent with the literature, no significant changes in volume or intensity were observed [32].

The athlete’s overall training strategy focused on endurance, prioritizing steady-state efforts, with limited and unstructured sprint or speed sessions. A large number of sessions included small amounts of both MIT (<10 min) and HIT (<6 min), likely due to the stochastic nature of the terrain and power output or the unstructured sessions that included brief “intensity moments”. Including these moments in the overall TID calculation is fundamental to avoid losing the chronic impact of cumulative training. Over the analyzed period, the quantity and distribution of both MIT and HIT progressively changed. Initially, volume and intensity were performed in separate sessions, but the athlete gradually shifted to a more integrated structure, combining high-volume and high-intensity days. This transition began with an increase in total HIT and culminated in the inclusion of intervals on high-volume days. Although the total amount of HIT remained consistent, it was progressively incorporated into long-ride days. The overall structure of the four years clearly reflects the concept of progressive overload, primarily driven by the accumulation of MIT and HIT and secondarily by the integration of more complex training sessions.

From a broader perspective, we can conclude that over these four years, no single training model—be it block, polarized, or pyramidal—was employed. Instead, a mixed approach was adopted and tailored to different periods, aligning with non-linear findings reported in other studies [33,34]. Based on the data and the athlete’s feedback, we can conclude that absolute power and various RPOs improved significantly over the first two years. In the latter two years, the most notable improvements were in durability and movement economy, as the athlete demonstrated an improved ability to sustain more efforts at the same power output within each session. As a further point, the gradual increase in training load observed in this case study is consistent with injury prevention principles, suggesting potential applications for safe exercise prescription in the general population [35].

## 5. Conclusions

This retrospective analysis of the preparation for the ZA finals of a contest winner highlights a clear hierarchy of training stimuli that can inform coaching practices. Emphasizing a progression from the ability to perform intense intervals to sustaining longer efforts and ultimately incorporating these into high-volume sessions appeared effective in this case through an integrated shift from separate LIT/MIT to “all-in days”. This structured approach underscores the importance of gradual load progression in athlete development. Furthermore, the cautious use of competitions aligns with recent trends, allowing the majority of the season to focus on structured training. Coaches and practitioners can apply these principles to optimize the development of endurance athletes aiming for high-level performance.

The preparation for the ZA finals demonstrated the pivotal role of training volume in the athlete’s development, confirming its foundational importance in endurance sports. The strategic balance between structured training and competition was critical to the athlete’s success, supporting the idea that a deliberate and progressive approach to load management is essential for sustained performance improvements. These findings reinforce the importance of tailoring training programs to the specific demands of high-level competition and suggest their effectiveness in athletes with suitable training and genetic profiles. 

## Figures and Tables

**Table 1:** Training characteristics of the 4 years antecedent ZA finals.

		2019	2020	2021	2022
General data					
	Volume (h)	701	934	869	746
	Maximum monthly volume (h)	87	93	80	78
	Minimum monthly volume (h)	3	52	42	24
Training organization					
	Two-session training days (n)	28	55	43	23
	Indoor sessions (n)	53	135	40	106
	Race days (n)	1	1	6	12
LIT					
	Volume (h)	608	838	778	647
	Maximum monthly volume (min)	87	93	80	78
	Minimum monthly volume (min)	3	52	42	24
	Monthly LIT % ± SD	89 ± 6.1	89.7 ± 2	89.6 ± 3	87.3 ± 4.7
MIT					
	Volume (h)	69	67	62	65
	Maximum monthly volume (min)	14	12	9	14
	Minimum monthly volume (min)	0	2	3	1
	Monthly MIT % ± SD	8.2 ± 4	7.1 ± 2.2	7.1 ± 2.3	8.2 ± 3.4
	Session with 1–10% MIT (n)	125	148	208	164
	Session with 10–20% MIT (n)	66	74	50	53
	Session with 20–30% MIT (n)	24	11	11	12
	Session with >30% MIT (n)	3	4	2	17
	MIT in training with <50 kJ/kg (min)	2972	3314	2726	2422
	MIT in training with >50 kJ/kg (min)	1247	750	947	1503
HIT					
	Volume (h)	24	28	29	35
	Maximum monthly volume (min)	6	5	4	5
	Minimum monthly volume (min)	0	1	1	0
	Monthly HIT % ± SD	2.7 ± 2	2.9 ± 1.2	3.2 ± 1.5	4.5 ± 2
	Session with 1–10% HIT (n)	104	128	91	81
	Session with 10–15% HIT (n)	17	17	20	20
	Session with 15–20% HIT (n)	5	4	5	12
	Session with >20% HIT (n)	1	3	4	18
	HIT in training with <50 kJ/kg (min)	1112	1415	1200	1306
	HIT in training with >50 kJ/kg (min)	337	209	453	746

## Data Availability

The data are accessible upon request to the authors.

## Abbreviations

The following abbreviations are used in this manuscript

ZA	Zwift Academy
LIT	low-intensity training
MIT	moderate-intensity training
HIT	high-intensity training
U23	Under23
WT	World Tour
LT1	First Lactate Threshold
LT2	Second Lactate Threshold
PI	polarization index
RPO	recorded power output
CPET	cardiopulmonary exercise testing
